# Infectious thrombosis of the superior sagittal sinus with subarachnoid hemorrhage: A case report

**DOI:** 10.1097/MD.0000000000033218

**Published:** 2023-03-10

**Authors:** Junxi Li, Shenjie Li, Junrong Zhang, Yuqi Wen, Ke Wang, Xingzhao Luan, Wei Xiang, Ligang Chen, Jie Zhou

**Affiliations:** a Department of Neurosurgery, The Affiliated Hospital of Southwest Medical University, Luzhou, China; b Sichuan Clinical Research Center for Neurosurgery, Southwest Medical University, Luzhou, China; c Academician (Expert) Workstation of Sichuan Province, Southwest Medical University, Luzhou, China; d Department of Neurosurgery, The General Hospital of Western Theater Command PLA, Chengdu, China; e Neurological Diseases and Brain Function Laboratory, The Affiliated Hospital of Southwest Medical University, Luzhou, China.

**Keywords:** case report, infectious stroke, subarachnoid hemorrhage, superior sagittal sinus thrombosis

## Abstract

Cerebral venous sinus thrombosis (CVST) represents 0.5% to 1% of all strokes. CVST can cause headaches, epilepsy, and subarachnoid hemorrhage (SAH). CVST is easily misdiagnosed because of the variety and non-specificity of symptoms. Herein, we report a case of infectious thrombosis of the superior sagittal sinus with SAH.

**Patient concerns:**

A 34-year-old man presented to our hospital with a 4-hour history of sudden and persistent headache and dizziness with tonic convulsions of the limbs. Computed tomography revealed SAH with edema. Enhanced magnetic resonance imaging showed an irregular filling defect in the superior sagittal sinus.

**Diagnoses:**

The final diagnosis was hemorrhagic superior sagittal sinus thrombosis and secondary epilepsy.

**Interventions:**

He was treated with antibiotic, antiepileptic, fluids to rehydrate, and intravenous dehydration.

**Outcomes:**

After treatment, the seizures did not recur and the symptoms were relieved. One month after the antibiotic treatment, the muscle strength of the patient’s right extremity was restored to level 5, and there was no recurrence of his neurological symptoms.

**Lessons:**

We describe a case of infectious thrombosis of the superior sagittal sinus manifested as SAH, which is easily misdiagnosed, especially when patients present with an infection. Clinicians must therefore take care during the diagnosis and selection of the treatment strategy.

## 1. Introduction

Cerebral venous sinus thrombosis (CVST) was first reported by Ribes in 1825, and is a special form of ischemic cerebrovascular disease accounting for 0.5% to 1% of all cerebrovascular diseases.^[1]^ In patients with CVST, the incidence of subarachnoid hemorrhage (SAH) was 1%.^[2]^ Herein, we present the case of a patient with infectious thrombosis of the superior sagittal sinus that manifested as SAH.

## 2. Case report

**Figure 1.**
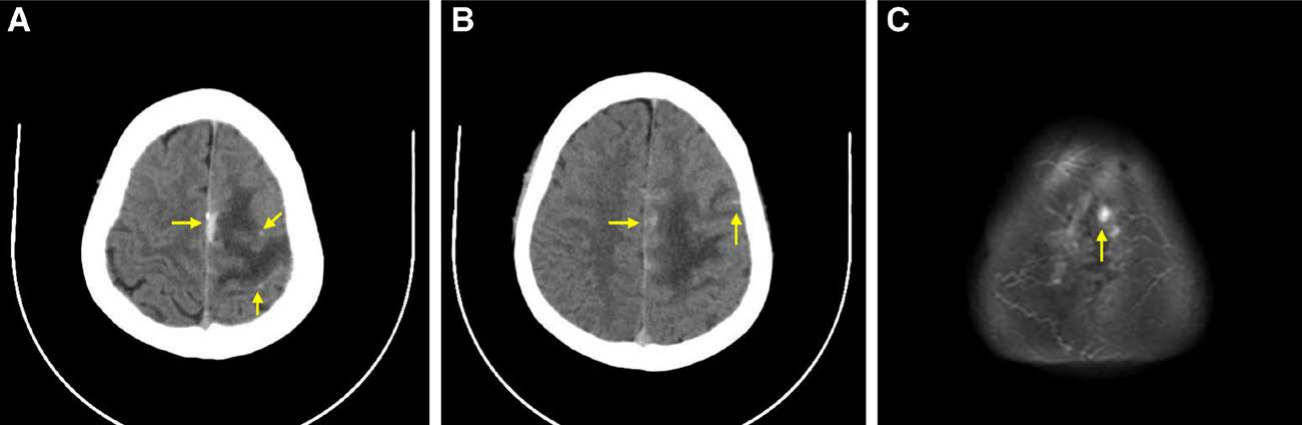
Images showed SAH and high signal in frontal bone plate. (A and B) CT scan showed left frontal lobe and interhemispheric cistern SAH with edema. (C) MRI showed high signal in left frontal bone plate on FLAIR. CT = computed tomography, FLAIR = fluid attenuated inversion recovery, MRI = magnetic resonance imaging, SAH = subarachnoid hemorrhage.

**Figure 2.**
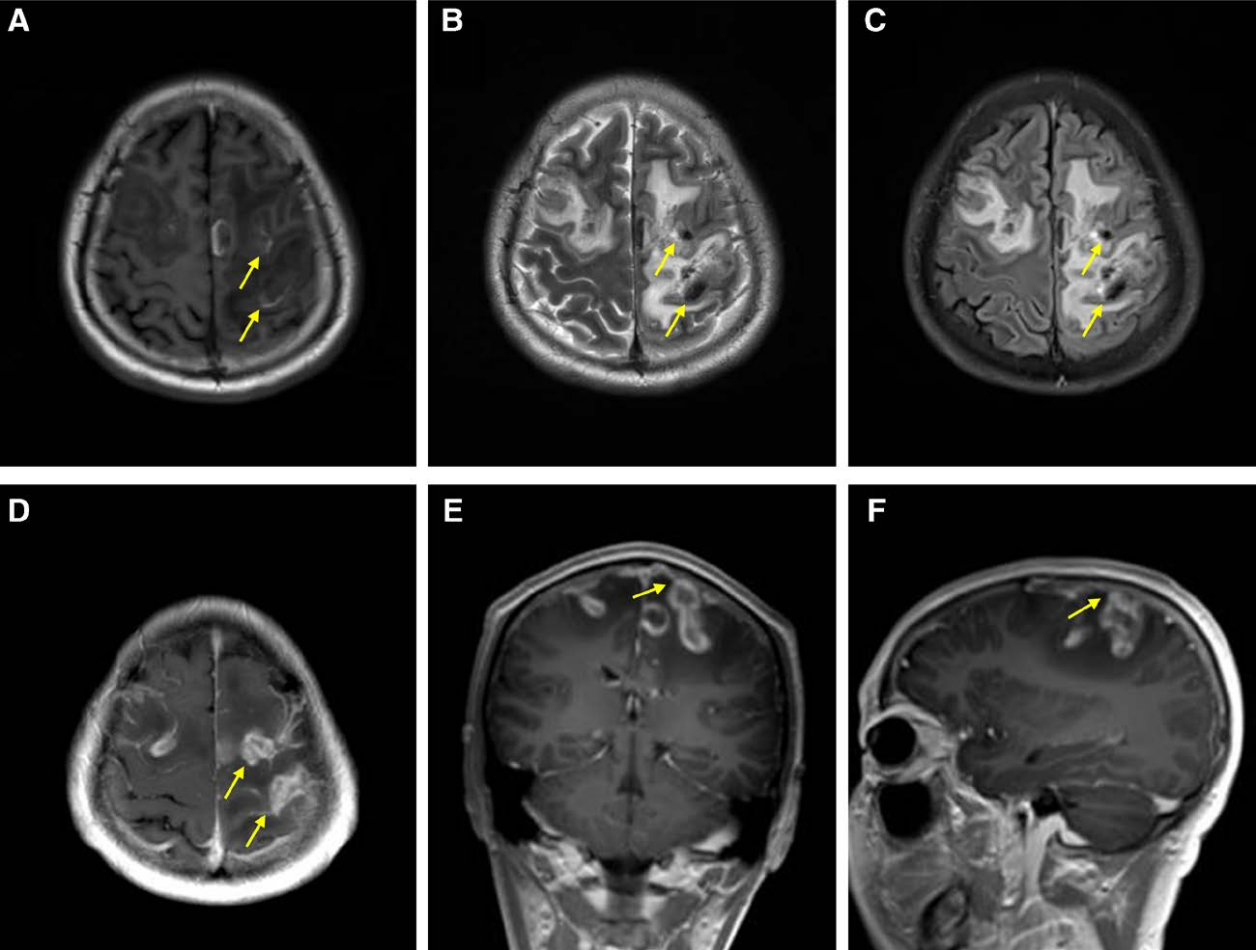
Images showed abscess was connected to the superior sagittal sinus. (A) MRI showed multiple ring-enhancing lesions on T1WI. (B) MRI showed multiple ring-hypointense lesions on T2WI. (C) MRI showed multiple ring-hypointense lesions on FLAIR. (D) Enhanced MRI showed multiple ring-enhancing lesions on T1WI. (E) Enhanced MRI showed abscess was connected to the superior sagittal sinus on T1WI coronary view. (F) Enhanced MRI showed abscess was connected to the superior sagittal sinus on T1WI sagittal view. MRI = magnetic resonance imaging, T1WI = T1-weighted imaging.

**Figure 3.**
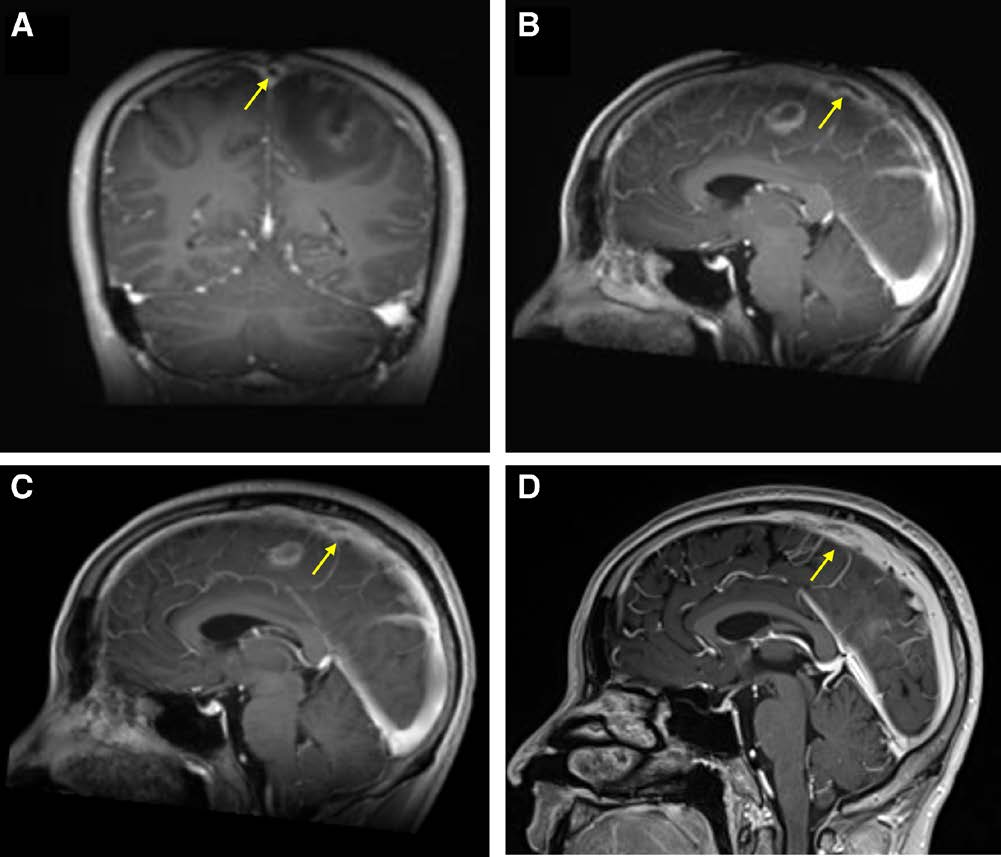
Images before and after the treatment. (A) Enhanced MRI showed irregular filling defect of middle superior sagittal sinus on T1WI coronary view before the treatment. (B) Enhanced MRI showed irregular filling defect of middle superior sagittal sinus on T1WI sagittal view before the treatment. (C) Enhanced MRI showed irregular filling defect of middle superior sagittal sinus on T1WI coronary view after the treatment. (D) Enhanced MRI showed irregular filling defect of middle superior sagittal sinus on T1WI sagittal view after the treatment. MRI = magnetic resonance imaging, T1WI = T1-weighted imaging.

A 34-year-old man presented to our hospital with a 4-hour history of sudden and persistent headache and dizziness with tonic convulsions of the limbs. He had 4 tonic seizures with impaired awareness and tonic convulsions of limbs. The patient had been taking thalidomide, sulfasalazine and meloxicam for ankylosing spondylitis, and his symptoms were moderate remission, and he had a history of ruptured pustulosis. The patient’s consciousness was greatly reduced (Glasgow Coma Scale score of 13), right upper limb muscle strength was level 3, right lower limb muscle strength was level 4 and left limbs muscle strength was level 4, and the meningeal irritation sign was positive. Laboratory studies showed that the white blood cell count was 11.78 × 10^9^/L, neutrophil percentage was 87.00%, and the percentage of lymphocytes was 8.40%. The cerebrospinal fluid (CSF) pressure for the lumbar puncture was 210 mm H_2_O and the CSF examination showed glucose concentration was 4.48 mmol/L, chlorine concentration was 132 mmol/L, protein concentration was 0.312 g/L and white blood cell-counting was 5 × 10^6^/L, and the CSF culture test was negative. The fibrin degradation product of 9.10 µg/mL (reference value, <5 µg/mL) and d-dimer of 3.43 µg/mL (reference value, <0.55 µg/mL). The computed tomography (CT) scan showed a left frontal lobe and interhemispheric cistern SAH with edema (Fig. 1A and B). Magnetic resonance imaging (MRI) showed a high signal in the left frontal bone plate on fluid attenuated inversion recovery (Fig. 1C). Multiple ring-enhancing lesions on T1-weighted imaging (T1WI) and Enhanced T1WI, multiple ring-hypointense lesions on T1WI and fluid attenuated inversion recovery were observed, suggesting intracranial infection and Enhanced MRI showed abscess was connected to the superior sagittal sinus on T1WI coronary and sagittal view, suggesting infection might cause thrombosis in the superior sagittal sinus (Fig. 2A–F). Irregular filling defect of the middle superior sagittal sinus, indicating thrombosis (Fig. 3A and B).

The patient was treated with valproate sodium as antiepileptic medication (20 mg/kg/d), mannitol dehydration treatment (1.25 g/kg/d), subcutaneous low molecular weight heparin (100 IU/kg/d), and ceftriaxone sodium as antibiotic treatments (67 mg/kg/d) for 1 week. On day 6 after treatment, the seizures did not recur, and the patient’s right upper extremity muscle strength was level 3, and the rest limbs muscle strength were level 5. Repeated enhanced MRI showed that the irregular filling defect of the superior sagittal sinus was smaller than before (Fig. 3C). After discharge, the patient was treated with oral ceftriaxone sodium (67 mg/kg/d) for 1 month, and levetiracetam (17 mg/kg/d), clopidogrel (1.25 mg/kg/d), rivaroxaban (0.17 mg/kg/d), and celecoxib (6.7 mg/kg/d) for 3 months.

One month after discharge, Enhanced MRI demonstrated that the filling defect in the superior sagittal sinus decreased (Fig. 3D). Outpatient follow-up showed that the muscle strength of the patient’s right limb was restored to level 5, and there was no recurrence of his neurological symptoms.

## 3. Discussion

To our knowledge, this is the first reported case of infectious thrombosis of the superior sagittal sinus with SAH due to the long-term used of immunosuppressants for ankylosing spondylitis. Although our patient had headache, epilepsy, SAH, and cerebral edema, we did not make a diagnosis of thrombosis of the superior sagittal sinus because these symptoms were nonspecific. Previous pointed out that up to 80% of CT studies were abnormal in CVST.^[3]^ But in our case, the CVST was not diagnosed based on the CT images. SAH and cerebral edema were the only abnormality on CT and we ruled out ruptured aneurysm by digital subtraction angiography. We finally diagnosed with thrombosis of the superior sagittal sinus based on the “empty delta sign” through enhanced MRI, patient’s history of ruptured pustulosis and the long-term used of immunosuppressants for ankylosing spondylitis.^[4]^ Therefore, the patient’s past medical history and imaging studies are very important to the diagnosis.

The major syndromes of thrombosis of the superior sagittal sinus are focal neurological abnormalities, isolated intracranial hypertension, encephalopathy, and seizures.^[5]^ However, SAH due to infectious thrombosis of the superior sagittal sinus is rare. SAH may have been caused by rupture of a dilated vein associated with thrombosis of the superior sagittal sinus in our case.^[6]^ Infection from the cortex can spread to the subperiosteal space (Fig. 1C), in which the veins can communicate with several emissary veins to the diploid vein of the skull and the intracranial dural sinus. As inflammation develops and superior sagittal sinus thrombosis leads to the production of various inflammatory factors and changes in vascular structure, leukocyte exudation increases, releasing inflammatory factors to change the blood composition to lead to a prothrombic state. The infection can also enter the subarachnoid space through arachnoid granulation, resulting in arachnoiditis. The pathogenesis of intracerebral hemorrhage may include vascular destruction and an inflammatory response in the subarachnoid space.^[7–10]^ In addition, ankylosing spondylitis is an autoimmune disease that also has a vascular autoimmune response which can cause interleukin-6, tumor necrosis factor-α and other inflammatory factors to increase, resulting in vascular immunological damage, atherosclerosis, abnormal coagulation, and cerebral thrombosis.^[11,12]^ In our case, long-term used of immunosuppressants for ankylosing spondylitis and had a history of ruptured pustulosis were risk factors for superior sagittal sinus thrombosis and probably also destroyed the vessel wall and caused vascular endothelial damage.^[5]^ We believe that our patient more likely developed thrombosis and SAH through the former mechanism.

In our case, the patient received ceftriaxone sodium as antibiotic treatment for 1 month, and oral clopidogrel and rivaroxaban as anticoagulant therapy for 3 months.^[13]^ After 6 days of treatment, the patient’s enhanced MRI showed that the irregular filling defect of the superior sagittal sinus was smaller than before and after a month of oral anticoagulant and antibiotic therapy, the patient recovered well and enhanced MRI demonstrated that the filling defect in the superior sagittal sinus significantly resolved. In cases of infectious thrombosis of the superior sagittal sinus with SAH, CT, and MRI play an important role in the diagnosis and efficacy evaluation of patients. Although the optimal duration of oral anticoagulant therapy and the time to the resolution of the thrombosis are unclear, early use of antibiotics and anticoagulation are necessary.^[13]^

In conclusion, infectious thrombosis of the superior sagittal sinus with SAH is rare and difficult to diagnose. We describe a case of infectious thrombosis of the superior sagittal sinus manifested as SAH. This case has a variety of symptoms making it prone to misdiagnosis, especially when patients present with an infection. Currently, CT, MRI, magnetic resonance angiography, etiological diagnosis and CSF examination are the main methods for diagnosing CVST. The combination of antibiotics and anticoagulation therapy can help cure the disease.^[3,13]^ Clinicians must therefore take care during the diagnosis and selection of the treatment strategy.

## Author contributions

**Conceptualization:** Junxi Li, Junrong Zhang.

**Data curation:** Shenjie Li.

**Methodology:** Junxi Li, Junrong Zhang, Yuqi Wen, Ke Wang, Wei Xiang.

**Supervision:** Ligang Chen, Jie Zhou.

**Validation:** Xingzhao Luan.

**Writing – original draft:** Junxi Li.
